# Historical Occurrence of Algal Blooms in the Northern Beibu Gulf of China and Implications for Future Trends

**DOI:** 10.3389/fmicb.2019.00451

**Published:** 2019-03-13

**Authors:** Yixiao Xu, Teng Zhang, Jin Zhou

**Affiliations:** ^1^Key Laboratory of Environment Change and Resources Use in Beibu Gulf, Ministry of Education, Guangxi Teachers Education University, Nanning, China; ^2^Guangxi Key Laboratory of Earth Surface Processes and Intelligent Simulation, Guangxi Teachers Education University, Nanning, China; ^3^Division of Ocean Science and Technology, Graduate School at Shenzhen, Tsinghua University, Shenzhen, China

**Keywords:** algal blooms, Beibu Gulf, occurrence trend, *Phaeocystis globosa*, marine pollution, management

## Abstract

Large-scale harmful algal blooms (HABs) occur in the coastal waters of the northern Beibu Gulf, China, and have deleterious effects on the marine ecosystem. The frequency, duration, and extent of HAB events in this region have increased over the last 30 years. However, the underlying causes of HABs and their likely future trends are unclear. To investigate, we evaluated historical data for temporal trends of HABs in the Beibu Gulf, and association with environmental factors as possible drivers. The results confirmed that HAB events had increased in frequency, from 6 reported events during the period 1985–2000, to 13 during 2001–2010, and 20 during 2011–2017. We also found that the geographic scale of algal blooms had increased from tens of km^2^ to hundreds of km^2^. There were temporal changes in HAB trigger species: prior to 2000, the cyanobacteria *Microcystis aeruginosa* was the dominant species, while during the period 2001–2010, blooms of cyanobacteria, dinoflagellates, and diatoms co-occurred, and during 2011–2017, the haptophyte *Phaeocystis globosa* became the dominant algal bloom species. Principal component analysis and variation partitioning analysis indicated that nutrient discharge, industrial development, and human activities were the key drivers of HAB events, and redundancy analysis showed that variation in the algal community tended to be driven by nutrient structure. Other factors, such as shipping activities and mariculture, also contributed to HAB events and algal succession, especially to *P. globosa* blooms. We speculated that the increasing severity of algal blooms in the northern Beibu Gulf reflects a more complex aquatic environment and highlights the damaging effects of anthropogenic inputs, urbanization development, and an expanding industrial marine-economy on the marine ecosystem. This research provides more insight into the increase of HABs and will aid their management in the Beibu Gulf.

## Introduction

Phytoplankton are a fundamental component of the marine ecosystem, playing multiple roles in nutrient cycling, and supporting global biological and geochemical processes (Lindh et al., 2013). However, excessive propagation of phytoplankton may cause an ecological phenomenon called harmful algal blooms (HABs). Some algal species that cause HABs produce toxins which lead to the human poisoning syndromes called paralytic shellfish poisoning, diarrhetic shellfish poisoning, amnesic shellfish poisoning, neurotoxic shellfish poisoning, and ciguatera fish poisoning. In other cases, non-toxic, high-biomass HABs can kill fish and marine organisms through both physical and chemical mechanisms, and can also induce hypoxia or anoxia, killing marine life at multiple trophic levels (Heisler et al., 2008).

HABs have significantly increased throughout the world's coastal oceans over the last century mostly due to seawater eutrophication and climate change (Anderson et al., 2012; Glibert et al., 2018). China is also challenged by eutrophication and recurrent, large, and diversified algal blooms including red tides, green tides, and brown tides in coastal waters (Yu and Liu, 2016; Yi et al., 2018). The most persistent and damaging HABs occur in the Bohai Sea, Changjiang River estuary, and the coastal waters of the South China Sea (Tang et al., 2006; Yu and Liu, 2016; Zhou et al., 2017).

The semi-enclosed body of water of the northern Beibu Gulf is in southwest China, northwest of the South China Sea. Algal blooms in the region are fairly new to scientists and the public, as it is often thought of as the last “clean ocean” and one of the most abundant fishing grounds in Chinese coastal waters (Chen et al., 2011; Zhong, 2015). It was thus a shock to the local government and even to HAB scientists when *Phaeocystis globosa* blooms almost blocked the cooling waters used for a nuclear power station in Fangchenggang (Cao et al., 2017; Gong et al., 2018), covered waters near the nuclear station, and broke out simultaneously across the whole coast of the northern Beibu Gulf (Kaiser et al., 2013; Lai et al., 2014; Luo et al., 2016). Against this background, our study reviewed algal blooms from 1980s to 2017, and analyzed their frequency, duration, geographical distribution, and associated physicochemical parameters in the Beibu Gulf, and aimed to speculate on future trends in HABs occurrence to improve mitigation and management of future HABs in the region.

## Materials and Methods

### Study Area

**Figure 1:**
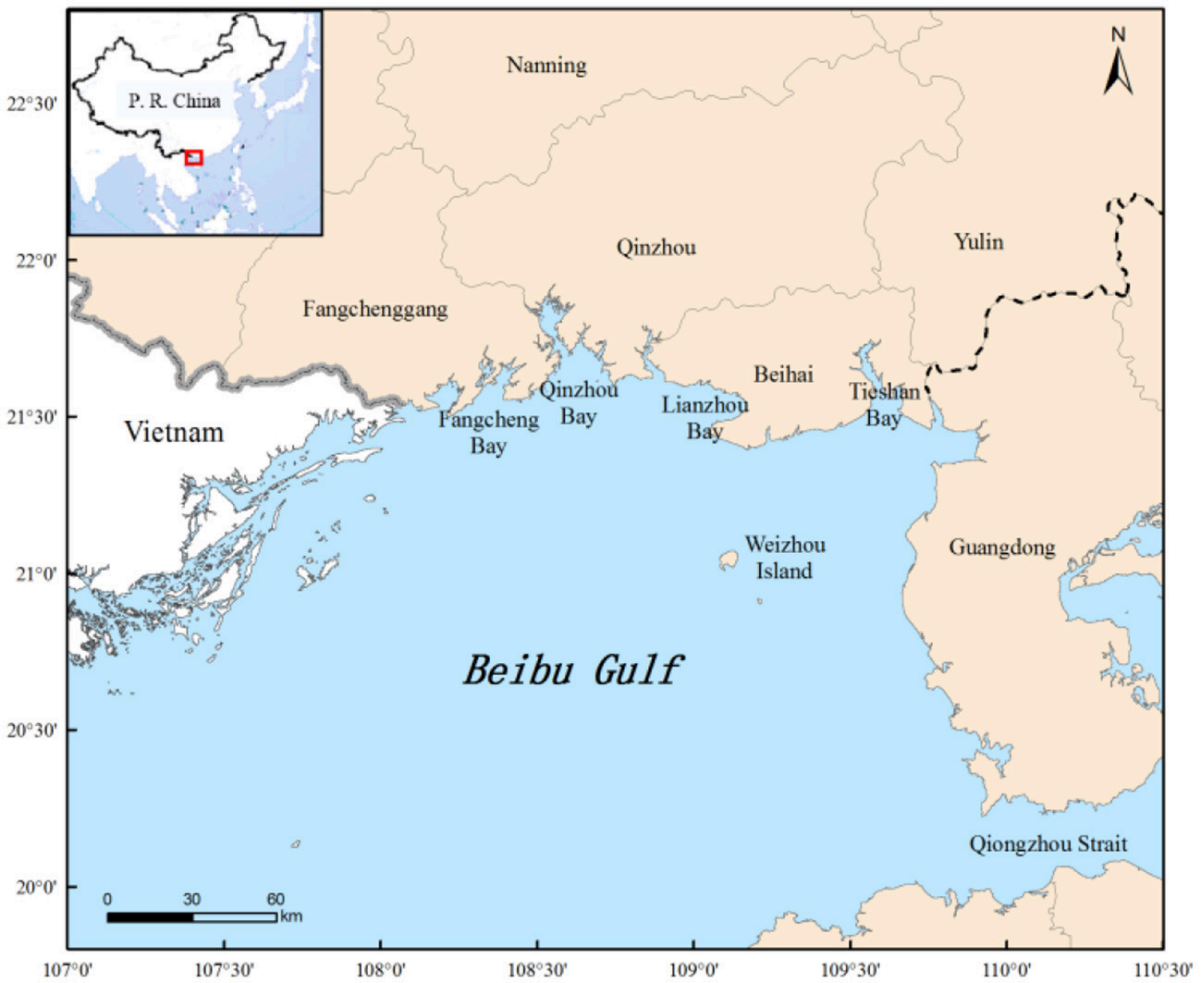
Location of the study area.

The northern Beibu Gulf (20° 58′-22° 50′ N, 107° 29′-110° 20′ E) (Figure 1) covers an area of 130,000 km^2^, with an average water depth of 38 m and a 1,629 km coastline (Chen et al., 2011; Li et al., 2014b). Tieshan, Lianzhou, Qinzhou, Fangchenggang, and Zhenzhu bays lie from East to West, with Weizhou Island offshore (Figure 1). The climate is tropical monsoon, with prevailing southwest winds in summer and northeast winds in winter, and an average annual seawater temperature of 24.5°C. The warm wet weather, in conjunction with abundant nutrient input from coastal rivers, such as the Nanliujiang and Qinjiang rivers, creates one of the most abundant fishing grounds in China (Chen et al., 2011).

### Data

Algal blooms recorded between 1985 and 2017 in the northern Beibu Gulf were collected from published literature, online media, monitoring station reports, and personal observations. Information including location, causative organism, taxonomy, and bloom area were extracted from these raw algal bloom data and are summarized in Table 1. Data sources are summarized in Table 2 and are provided as Supplements 1–15: (1) population; (2) gross industrial output value; (3) gross domestic product; (4) consumption of chemical fertilizers; (5) Guangxi seawater cultured areas; (6) Guangxi artificially cultured products; (7) Guangxi seawater aquatic products; (8) Guangxi marine related GDP; (9) fishing boats in the northern Beibu Gulf of China from 2001 to 2010; (10–14) seawater nutrient content of NO_3_, NO_2_, NH_4_, PO_4_, S_i_O_3_, dissolved inorganic nitrogen (DIN), and Chemical Oxygen Demand (COD) in Tieshan Bay from 1983 to 2012, in Qinzhou Bay, Fangcheng Bay and the northern Beibu Gulf of China from 1983 to 2015, and in Lianzhou Bay from 1991 to 2015; and (15) areas of five classification of summer seawater quality between 2010 and 2016 in the northern Beibu Gulf. Among them, (1), (4–9) were defined as human activity, (2,3) as industrial development, (5–8) as mariculture, and (10–15) as environmental factors.

**Table 1:** Details and sources of historical algal bloom data (1985–2017).

**Date**	**Location**	**Causative organism**	**Taxonomy**	**Area (km^**2**^)**	**References**
June 1985	Weizhou Island	*Trichodesmium erythraeum*	Cyanophyta, Cyanophyceae	—	Liang and Qian, 1991
March 1995	Lianzhou Bay	*Microcystis* sp.	Cyanophyta, Chroococcophyceae	≤10	Wei and He, 1998; Li et al., 2014a
Apr 1995	Beihai Port	*Microcystis aeruginosa*	Cyanophyta, Chroococcophyceae	—	Beihai Yearbook Editorial Committee, 1997
Nov 1995	Beihai Port	*Microcystis aeruginosa*	Cyanophyta, Chroococcophyceae	—	Beihai Yearbook Editorial Committee, 1997
Dec 1999	Weizhou Island	*Microcystis aeruginosa*	Cyanophyta, Chroococcophyceae	>50	Southland Morning Post, 2002; Qiu et al., 2005
May 2000	Weizhou Island	*Microcystis aeruginosa*	Cyanophyta, Chroococcophyceae	3	China Ocean Yearbook Editorial Committee, 2002
May 2001	Weizhou Island	—	—	20	China Ocean Yearbook Editorial Committee, 2003; Luo et al., 2016
May, 2002	Weizhou Island	—	—	5	Beihai Yearbook Editorial Committee, 2003; Marine Environmental Monitoring Center of Beihai Guangxi, 2006
June, 2002	Weizhou Island	*Trichodesmium hildebrandtii*	Cyanophyta, Cyanophyceae	20	Beihai Yearbook Editorial Committee, 2003
June 2003	Weizhou Island	*Microcystis flos-aquae*	Cyanophyta, Chroococcophyceae	20	Marine Environmental Monitoring Center of Beihai Guangxi, 2006
July 2003	Weizhou Island	*Trichodesmium erythraeum*	Cyanophyta, Cyanophyceae	10	China Ocean Yearbook Editorial Committee, 2004; China Oceanic Information Network, 2011
Feb 2004	Beihai	*Microcystis flos-aquae*	Cyanophyta, Chroococcophyceae	40	Marine Environmental Monitoring Center of Beihai Guangxi, 2006
Mar, 2004	Weizhou Island	*Microcystis flos-aquae*	Cyanophyta, Chroococcophyceae	2	Marine Environmental Monitoring Center of Beihai Guangxi, 2006
June 2004	Weizhou Island	*Trichodesmium erythraeum*	Cyanophyta, Cyanophyceae	40	Beihai Chorography Office, 2005; Li et al., 2009
May 2005	Weizhou Island	—	—	250	Tong, 2006
Apr 2008	Weizhou Island	*Noctiluca scintillans*	Dinophyta, Dinophyceae	0.025	State Oceanic Administration People's Republic of China, 2009
Apr 2008	Qinzhou Bay	*Noctiluca scintillans*	Dinophyta, Dinophyceae	0.0001	State Oceanic Administration People's Republic of China, 2009
July 2009	Lianzhou Bay	*Skeletonema costatum*	Bacillariophyta, Mediophyceae	—	Li et al., 2011
May 2010	Beibu Gulf	*Guinardia flaccida*	Bacillariophyta, Coscinodiscophyceae	150	Dou et al., 2015
Apr 2011	Qinzhou Bay	*Noctiluca scintillans*	Dinophyta, Dinophyceae	1.2	Southland Morning Post, 2015; Luo et al., 2016
Nov 2011	Beihai, Qinzhou, Fangcheng	*Phaeocystis globosa*	Haptophyta, Prymnesiophyceae	10	News China, 2011; Beihai Chorography Editorial Committee, 2012
Feb, 2012	Lianzhou Bay	*Guinardia flaccida*	Bacillariophyta, Coscinodiscophyceae	—	Li et al., 2015b
Jan–Dec 2013	Beibu Gulf (12 small blooms)	—	—	—	Southland Morning Post, 2015
Feb–Mar 2014	Tieshan Port-Shatian Port	*Phaeocystis globosa*	Haptophyta, Prymnesiophyceae	—	Li et al., 2015a
Feb–Mar 2014	Lianzhou Bay- Beihai	*Phaeocystis globosa*	Haptophyta, Prymnesiophyceae	—	Li et al., 2015a
Jan 2015	Almost entire northern Beibu Gulf coast	*Phaeocystis globosa*	Haptophyta, Prymnesiophyceae	—	Southland Morning Post, 2015
Feb 2017	Qinzhou Bay	*Phaeocystis globosa*	Haptophyta, Prymnesiophyceae	—	Author's observation
Mar 2017	Almost entire Weizhou Island coast	*Phaeocystis globosa*	Haptophyta, Prymnesiophyceae	—	Author's observation

**Table 2:** Supplements, data sources, authors and data for (1) population, (2) gross industrial output value, (3) gross domestic product, (4) consumption of chemical fertilizers, (5) Guangxi seawater cultured areas, (6) Guangxi artificially cultured products, (7) Guangxi seawater aquatic products, (8) Guangxi marine related GDP, (9) Fishing boats in the northern Beibu Gulf of China from 2001 to 2010, (10–14) seawater nutrient content of NO_3_, NO_2_, NH_4_, PO_4_, S_i_O_3_, dissolved inorganic nitrogen (DIN), and Chemical Oxygen Demand (COD) in Tieshan Bay, Lianzhou Bay, Qinzhou Bay, Fangcheng Bay and the northern Beibu Gulf of China from 1983 to 2015, and (15) areas of five classification of summer seawater quality between 2010 and 2016 in the northern Beibu Gulf.

**Supplements**	**Data sources**	**Authors**	**Data**
Supplement 1	1989–2016 Guangxi Statistical Yearbook	China Statistics Press	Population (10^4^ persons) for Nanning, Beihai, Qinzhou, and Fangchenggang from 1988 to 2015.
Supplement 2	1989–2016 Guangxi Statistical Yearbook	China Statistics Press	Gross industrial output value (10^8^ Yuan) for Nanning, Beihai, Qinzhou, and Fangchenggang from 1988 to 2015.
Supplement 3	1989–2016 Guangxi Statistical Yearbook	China Statistics Press	Gross domestic product (10^8^ Yuan) for Nanning, Beihai, Qinzhou and Fangchenggang from 1988 to 2015.
Supplement 4	1989–2016 Guangxi Statistical Yearbook	China Statistics Press	Consumption of chemical fertilizers (10^4^ tons) for Nanning, Beihai, Qinzhou, and Fangchenggang from 1988 to 2015.
Supplement 5	1994–2007 Guangxi Statistical Yearbook	China Statistics Press	Guangxi seawater cultured areas (10^3^ hectares) from 1978 to 2006.
Supplement 6	1994–2016 Guangxi Statistical Yearbook	China Statistics Press	Guangxi artificially cultured products (10^4^ tons) from 1978 to 2015.
Supplement 7	1992–2017 Guangxi Statistical Yearbook	China Statistics Press	Guangxi seawater aquatic products (10^4^ tons) from 1978 to 2015.
Supplement 8	2007–2016 Guangxi Marine Economic Statistics Bulletin	Department of Ocean and Fisheries of Guangxi Zhuang Autonomous Region	Guangxi marine related GDP (10^9^ Yuan) from 2007 to 2016.
Supplement 9	Scientific publication	(Lan and Li, 2013)	Fishing boats in the northern Beibu Gulf of China from 2001 to 2010.
Supplement 10	Scientific publication	See Supplementary Table 1	Seawater nutrient content in Tieshan Bay, Guangxi from 1983 to 2012.
Supplement 11	Scientific publication	See Supplementary Table 2	Seawater nutrient content in Lianzhou Bay, Guangxi from 1991 to 2015.
Supplement 12	Scientific publication	See Supplementary Table 3	Seawater nutrient content in Qinzhou Bay, Guangxi from 1983 to 2015.
Supplement 13	Scientific publication	See Supplementary Table 4	Seawater nutrient content in Fangcheng Bay, Guangxi from 1983 to 2015.
Supplement 14	Scientific publication	See Supplementary Table 5	Seawater nutrient content in the northern Beibu Gulf, China from 1983 to 2015.
Supplement 15	2014-2016 Guangxi Marine Environmental Quality Bulletin	Department of Ocean and Fisheries of Guangxi Zhuang Autonomous Region	Areas of five classification of summer seawater quality between 2010 and 2016 in the northern Beibu Gulf (km^2^).

The seawater quality data containing the five classifications of summer seawater quality from 2010 to 2016 came from the 2014–2016 marine environmental quality bulletin of Guangxi, released by the Department of Ocean and Fisheries of the Guangxi Zhuang Autonomous Region. It was examined and classified according to the seawater quality standard of China (GB3097-1997), and was categorized from I to IV, where Grade I is defined as high quality seawater for ocean fishing, and marine nature reserves that contain rare and endangered marine organisms. Grade II is used for aquaculture, bathing beaches, and seawater activity areas with direct human contact, and also for industrial water use in relation to human consumption. Grade III is defined as general industrial water and coastal scenic areas, while Grade IV represents the worst quality seawater used for harbor and ocean engineering operations.

### Statistical Analysis

We used one-way ANOVA in SPSS (v. 13.0) (Chicago, Illinois, USA) to evaluate differences in environmental parameters during three time periods (1985–2000; 2001–2010; and 2011–2017) at the *p* < 0.05 or *p* < 0.01 level. Correlations between HAB outbreaks and physicochemical factors based on euclidean distance were calculated using principal component analysis and visualized using R software (v2.15.1, www.r-project.org). The relationship between the environmental factors, human activities, and HAB events from 1985 to 2017 was measured using multivariate correlation analysis (redundancy analysis), which was performed using the Canoco software package for Windows (v4.5) (Ithaca, New York, USA) with Monte Carlo permutation tests (499 permutations) (Terbraak and Smilauer, 2002; Legendre et al., 2011). HAB-causing species and environmental parameters used in the redundancy analysis were normalized through a logarithmic transformation (log_10_ (n+1)). We used variation partitioning analysis (VPA) to analyze the relative contribution of multiple parameters (industrial development, ID; environmental factors, EF; and human activities, HA) to HAB variation. The data came from Table 2 as defined in Data section. These data were (log_2_ (x+1)) transformed for standardization. VPA was performed using the VEGAN package in R.

## Results

### Historical HAB Events

**Figure 2:**
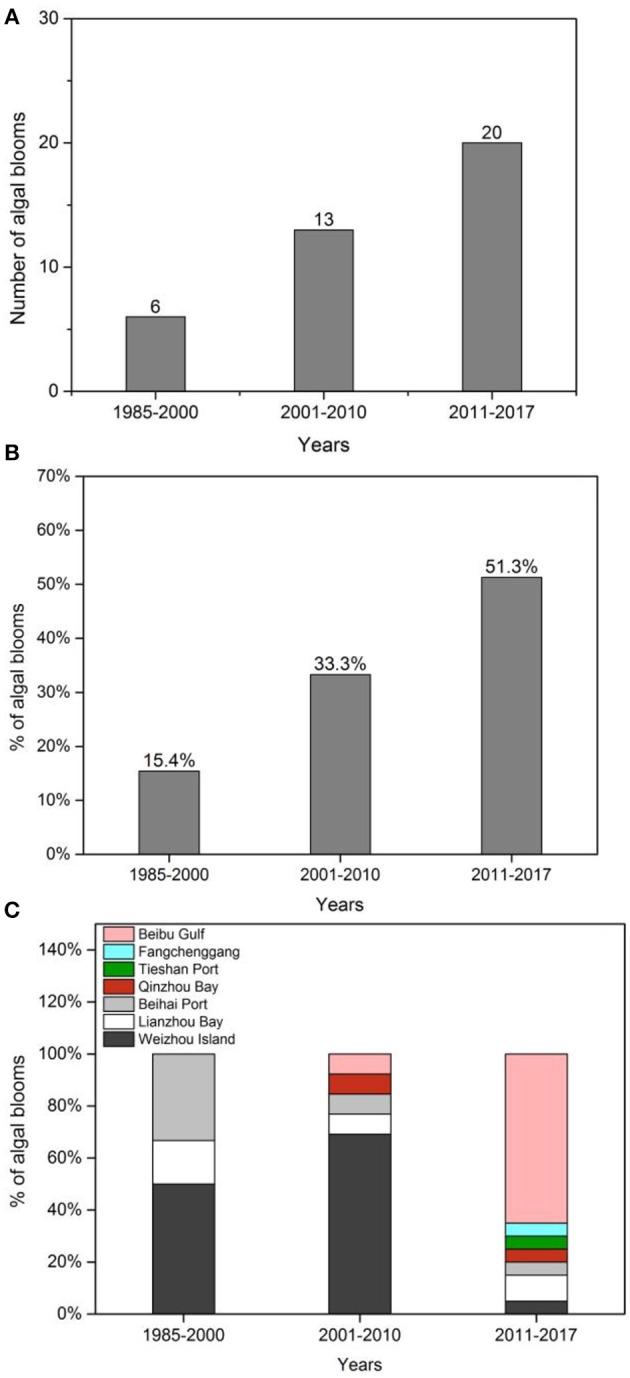
Number **(A)** and proportion (%) **(B)** of algal blooms reported in the northern Beibu Gulf of China from 1985–2017; **(C)** proportion (%) of algal blooms at different locations in the northern Beibu Gulf of China from 1985 to 2017.

There were 39 algal bloom events from 1985 to 2017, 15.4% occurred during the period 1985–2000, 33.3% during 2001–2010, and 51.3% during 2011–2017 (Figures 2A,B). The maximum area of algal blooms increased from >50 km^2^ in the 1990s to 250 km^2^ in the 2000s, and then HABs covered almost the entire coast between 2011 and 2017 (Table 1). Unfortunately, the area values between 2012 and 2017, when HABs became widespread, are not available. HAB duration was relatively short, occurring over days during the period 1985–2000; however, they typically occurred for weeks in the latter periods (Table 1).

Prior to 2000, HABs tended to occur at Beihai Port, Lianzhou Bay, and Weizhou Island; during the period 2001–2010, 70% of HABs occurred at Weizhou Island; and after 2011, HABs occurred at a greater number of sites, including along the coasts of Tieshan, Beihai, Qinzhou, Fangchenggang, and Weizhou Island (Figure 2C).

The causative microalgae included the cyanobacteria *Trichodesmium erythraeum* and *Microcystis* spp., with *M. aeruginosa* as the dominant species during the period 1985–2000. Blooms of cyanobacteria *T. hildebrandtii, T. erythraeum*, and *M. flos-aquae*, dinoflagellate *Noctiluca scintillans*, and diatoms *Skeletonema costatum* and *Guinardia flaccida* co-occurred without an obviously dominant species for the period 2001–2010. Between 2011 and 2017, the haptophyte *P. globosa* was the major species in blooms, particularly in the past 5 years (Table 1). We noticed that 4 of the 39 algal bloom events were associated with mortality of marine organisms such as fish and shrimp, and were attributed to *T. erythraeum* blooms in May 2001, July 2003, and June 2004 around Weizhou Island, and a *N. scintillans* bloom in Qinzhou in April 2011 (China Ocean Yearbook Editorial Committee, 2003, 2004; Beihai Chorography Office, 2005; State Oceanic Administration People's Republic of China, 2009; Luo et al., 2016).

### Industrial Development and Human Activities

**Figure 3:**
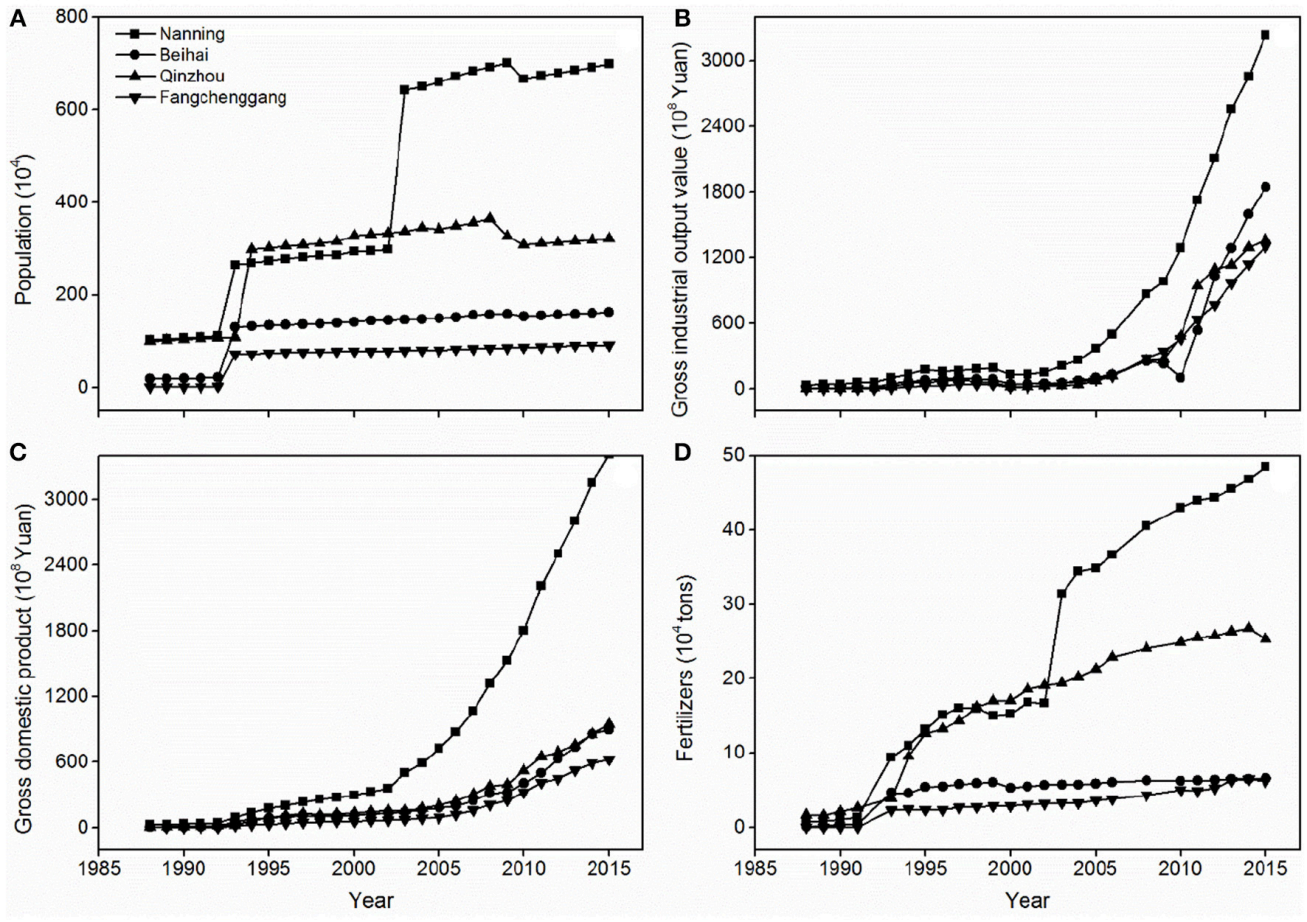
Time-series plots of **(A)** population (10^4^), **(B)** gross industrial output value (10^8^ Yuan), **(C)** gross domestic product (10^8^ Yuan), and **(D)** fertilizers (10^4^ tons) in four key regions of the Guangxi Beibu Gulf Economic Zone.

Nanning, Qinzhou, Beihai, and Fangchenggang are the main districts in the Beibu Gulf Economic Zone. During the period 1980s−2015, across the districts there were 3.2–68.0, 98.1–22,703.3, 113.4–868.0, and 15.2-20,733.3-fold increases in population, gross industrial output value, gross domestic product, and fertilizer, respectively (Figures 3A–D). Nevertheless, these parameters were quite stable until the 1990s, when the substantial increases started. Although Nanning and Qinzhou generally reached much higher values than Beihai and Fangchenggang since the 2000s, all maximum fold increases were found in Fangchenggang due to its small initiating values in the 1980s.

**Figure 4:**
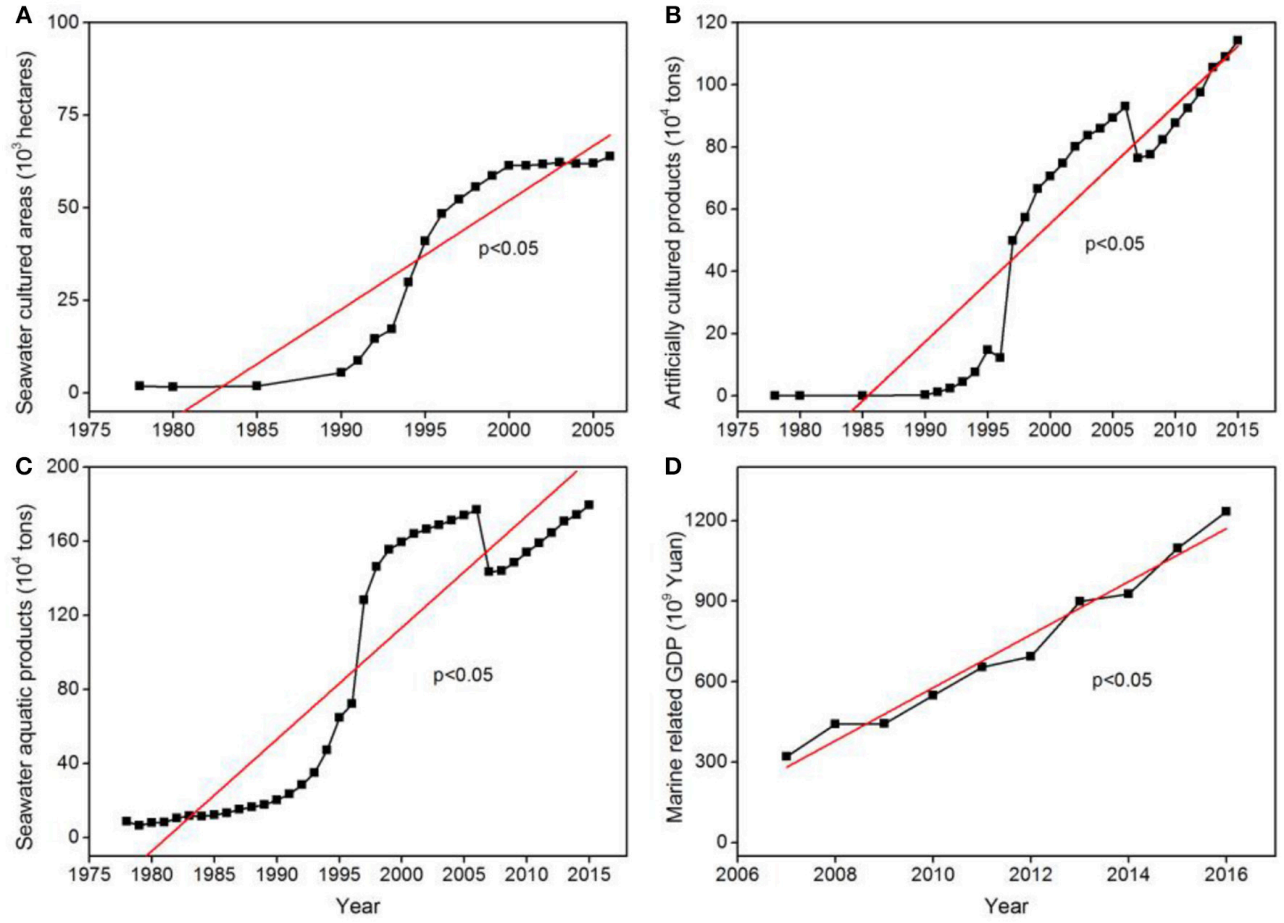
Time-series plots of **(A)** seawater cultured areas (10^3^ hectares), **(B)** artificially cultured products (10^4^ tons), **(C)** seawater aquatic products (10^4^ tons), and **(D)** marine related GDP (10^9^ Yuan) in the northern Beibu Gulf. Red line denotes change in trend. For p < 0.05, at the 0.05 level, the slope is significantly different from zero; for *p* > 0.05, at the 0.05 level, the slope is not significantly different from zero.

**Figure 5:**
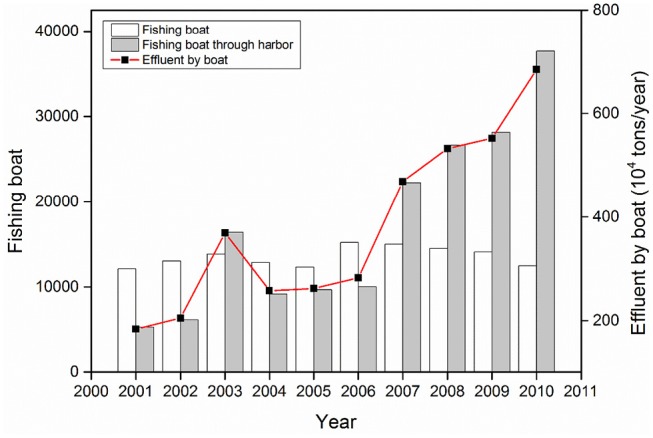
Number of fishing boats and harbored fishing boats, and amount of effluent produced by boat in the northern Beibu Gulf of China from 2001 to 2010.

The seawater cultured area increased 33.6-fold from 1978 to 2006; artificially cultured products and seawater aquatic products increased by 2,607.9- and 21.7-fold, respectively, from 1978 to 2015 (Figures 4A–C), with these parameters exhibiting a significant increase after the 1990s. There was an associated 3.9-fold increase in marine related GDP from 2007 to 2016 across the region (Figure 4D). Shipping transport in the northern Beibu Gulf intensified, with increases in the number of fishing boats (from 500 in 2001 to 3,850 in 2010) and effluent (3.74-fold increase from 2001 to 2010) (Figure 5).

### Environmental Factors

**Figure 6:**
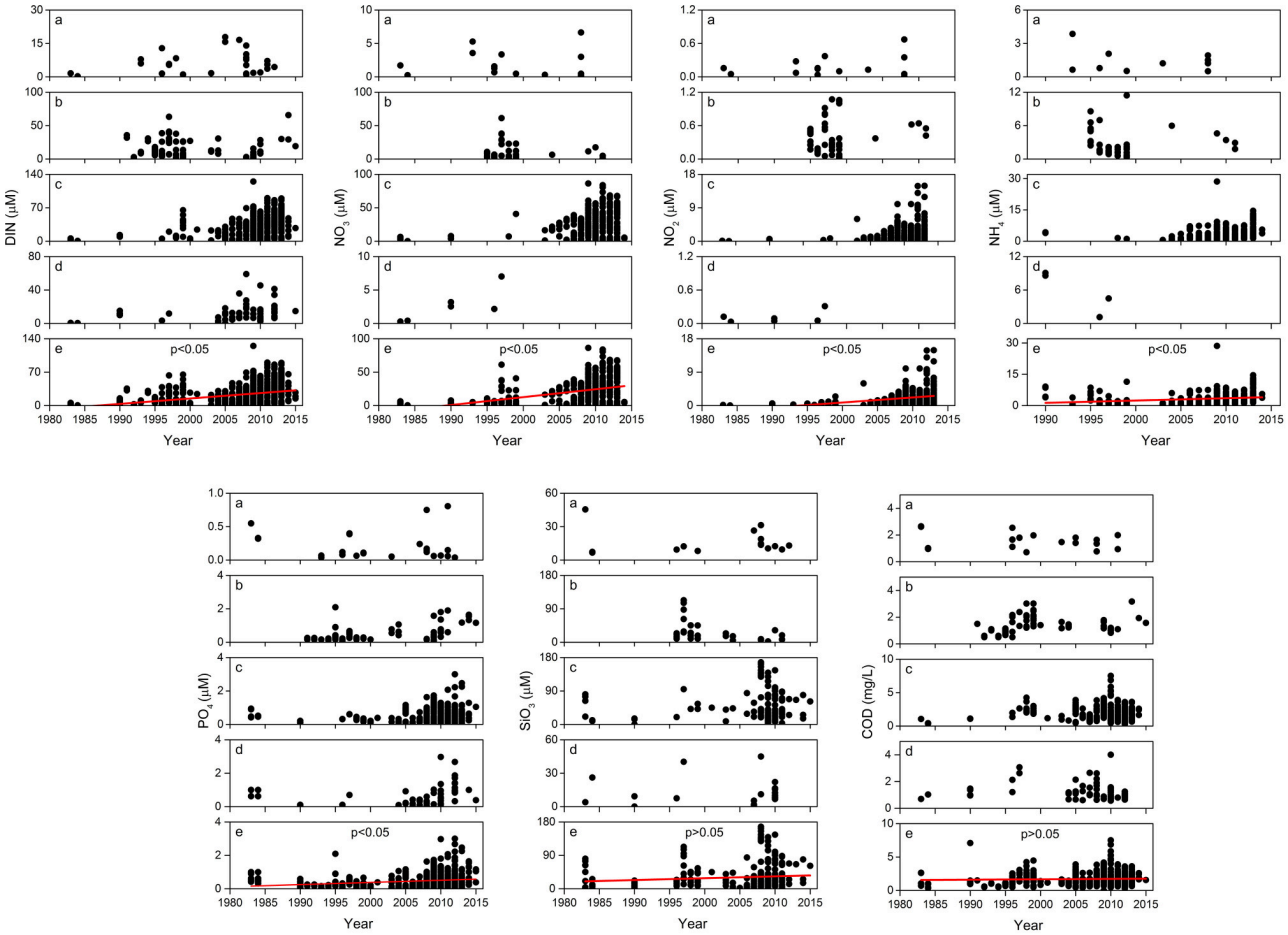
Temporal changes in seawater nutrient content between 1980 and 2015 in (a) Tieshan Bay; (b) Lianzhou Bay; (c) Qinzhou Bay; (d) Fangcheng Bay; and, (e) Beibu Gulf, where *n* = 697, 456, 453, 442, 708, 278, and 560 for linear fit of DIN, NO_3_, NO_2_, NH_4_, PO_4_, SiO_3_, and COD, respectively, and the red line denotes the long term trend. For *p* < 0.05, at the 0.05 level, the slope is significantly different from zero; for *p* > 0.05, at the 0.05 level, the slope is not significantly different from zero.

We found significant increases in the levels of DIN, NO_3_, NO_2_, NH_4_, and PO_4_ (*p* < 0.05) in the study region (Figure 6). From 1980 to 2010, there were 17.9-, 12.7-, 33.5-, and 1.5-fold increases in average DIN, NO_3_, NO_2_, and NH_4_ in the northern Beibu Gulf, respectively (Table 3). From 1990 to 2010, average PO_4_ increased almost 2-fold, and there were no significant differences in concentrations of SiO_3_ and COD (*p* > 0.05), although both were lowest in the 1980s (Figure 6, Table 3). Maximum concentrations of SiO_3_ in the northern Beibu Gulf increased from 81.0 μM in the 1980s to 113.0 μM in the 1990s, and to 167.1 μM in the 2000s, while maximum COD increased from 2.7 mg/L in the 1980s to 7.5 mg/L in 2010s (Figure 6).

**Table 3:** Long term trend in seawater nutrient (μM) and COD (mg/L) content in the northern Beibu Gulf. Data are means ±SD.

**Year**	**DIN**	**NO_**3**_**	**NO_**2**_**	**NH_**4**_**	**PO_**4**_**	**SiO_**3**_**	**COD**
1980s	1.7 ± 1.9	2.1 ± 2.4	0.07 ± 0.05	1.5 ± 0.4	0.6 ± 0.2	23.5 ± 28.5	1.2 ± 0.9
1990s	14.2 ± 13.8	9.3 ± 11.9	0.37 ± 0.39	2.7 ± 2.5	0.3 ± 0.3	28.2 ± 27.5	1.8 ± 1.0
2000s	19.4 ± 17.8	20.4 ± 16.5	1.72 ± 1.66	2.7 ± 2.8	0.4 ± 0.4	31.4 ± 38.6	1.8 ± 1.0
2010s	29.9 ± 21.9	26.5 ± 20.2	2.43 ± 2.55	4.0 ± 2.9	0.6 ± 0.5	33.6 ± 29.6	1.6 ± 1.0

**Figure 7:**
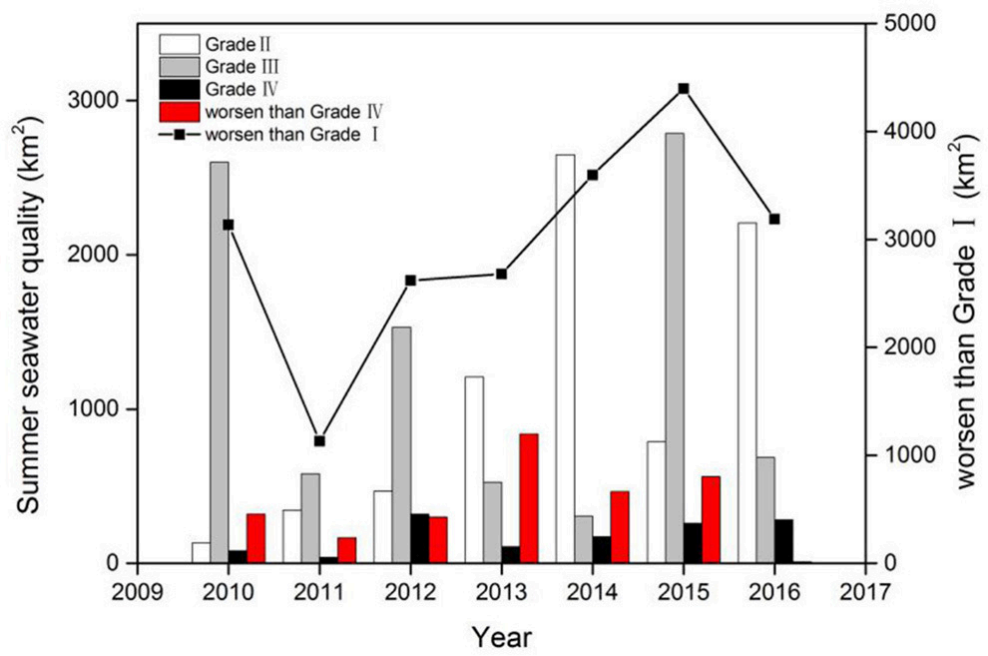
Summer seawater quality between 2009 and 2016 in the northern Beibu Gulf. Seawater quality is categorized from I–IV, where Grade I is defined as high quality seawater for ocean fishing, marine nature reserves that contain rare and endangered marine organism, while Grade IV represent the worst quality seawater used for harbor and ocean engineering operations.

There was a deterioration in summer seawater quality between 2009 and 2016, with a generally increasing trend in the area of seawater with quality ranked in the lowest category (Grade IV), quality worse than Grade IV, and quality lower than Grade I during 2011–2015 (Figure 7).

### Correlation Between Influencing Factors and HAB Events

**Figure 8:**
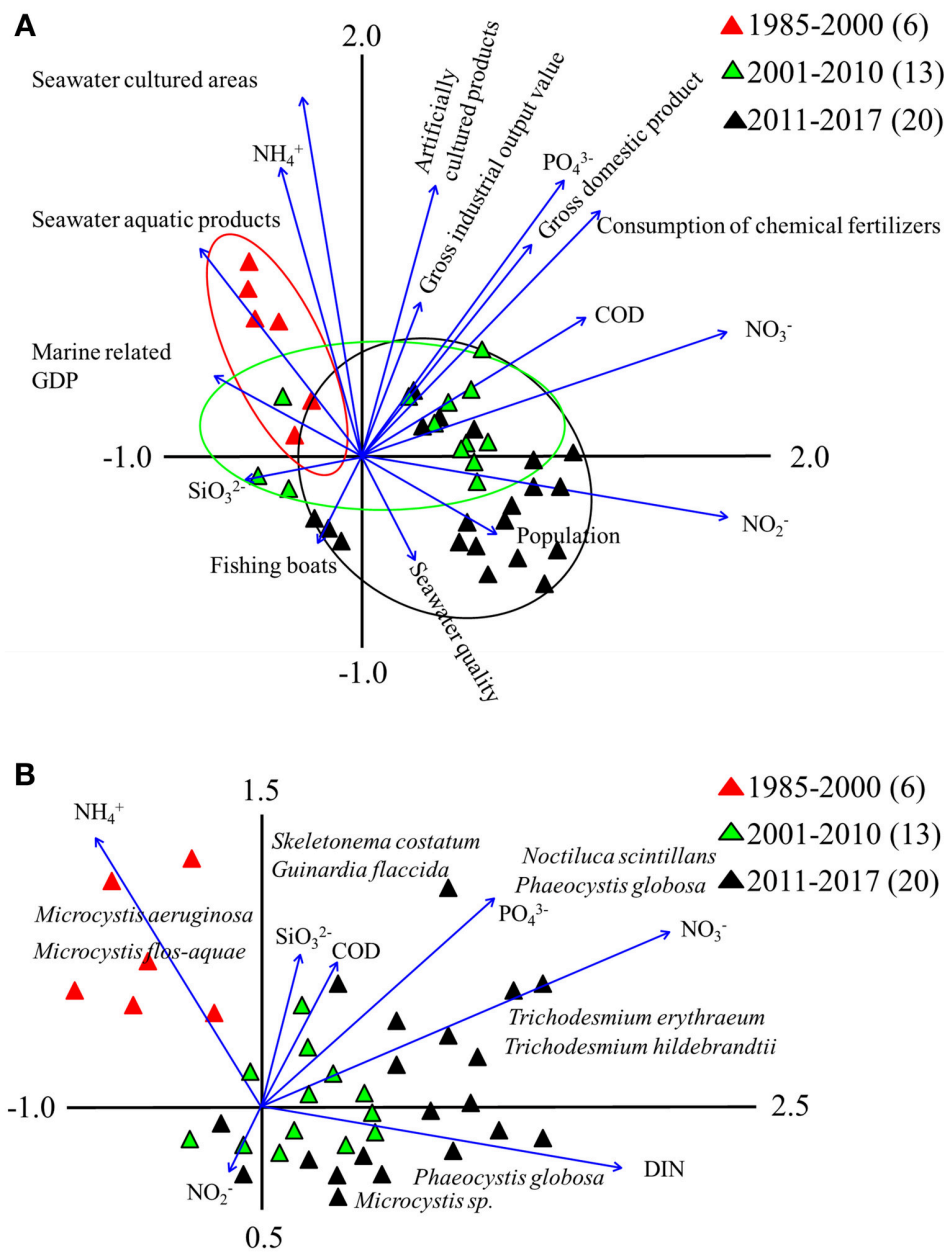
Principal component analysis **(A)** and redundancy analysis **(B)** of industrial development, environmental factors, human activities and HAB events at sampling stations from 1985 to 2017 in the study region. Arrow length indicates importance of each factor in HAB events.

We found there were temporal trends in multiple factors influencing HAB events (Figure 8A): between 1985 and 2000, there was an association with ${\text{NH}}_{4}^{+}$, seawater aquatic products, and marine related GDP value; while between 2001 and 2010, there was an association with artificially cultured products, fertilizer use, gross domestic product, and C-N-P-Si sources (such as COD, NO_3_, PO_4_, and SiO_3_). Between 2011 and 2017 there was an association between HAB events and NO_3_, NO_2_, COD, PO_4_, fishing activities, seawater quality, population number, industrial development, and mariculture.

For simplification, we used nutrients as the target in the redundancy analysis, and found that there was temporal variation in the dominant phytoplankton species (Figure 8B). Succession of algae tended to be driven by N and P content, and the two dominant species in earlier years, *M. aeruginosa* and *M. flos-aquae*, showed a preference for high levels of NH_4_. Two species prominent from 2001 to 2010, *S. costatum* and *G. flaccida*, were closely associated with SiO_3_ and COD. Other dominant species, *Trichodesmium* spp. and *N. scintillans* showed association with high levels of NO_3_ and PO_4_, and we found that DIN and PO_4_ influenced *P. globosa* abundance.

**Figure 9:**
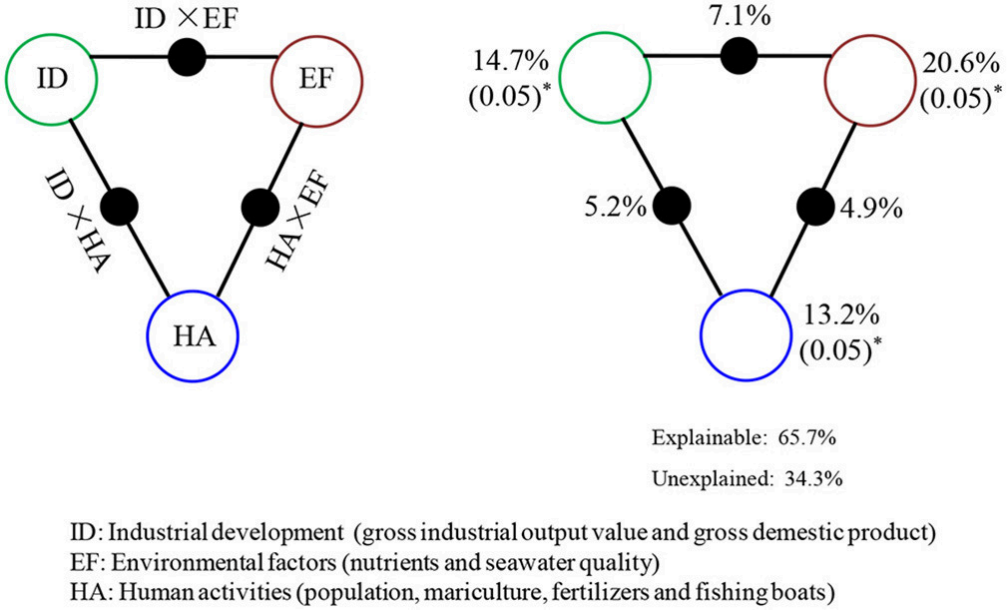
Variation partitioning analysis (VPA) of HAB events explained by multiple influencing factors including industrial development, ID; environmental factors, EF; and human activities, HA. The angles and sides of the triangles represent the variation explained by each single factor and by the combination of any two factors, respectively.

VPA showed that when combined, ID, EF, and HA explained 65.7% of the variation in HAB outbreaks (Figure 9), whereas independently, ID, EF, and HA explained 14.7% (*p* < 0.05), 20.6% (*p* < 0.05), and 13.2% (*p* < 0.05) of the variation, respectively. Interactions between ID and EF, EF and HA, and HA and ID explained 7.1, 4.9, and 5.2% of variation, respectively. Overall 34.3% of variation in HAB events was not explained by these parameters.

## Discussion

Similar to many coastal regions globally, HABs in the northern Beibu Gulf have significantly increased in frequency, duration, geographical distribution, and degree of harm in recent decades (Anderson et al., 2002; Heisler et al., 2008). However, compared with already developed Chinese coastal areas such as the Bohai estuary and Changjiang River Estuary, there was an ~ 10–15 year lag for the remarkable breakout of HABs in the northern Beibu Gulf (Luo et al., 2016; Yu and Liu, 2016; Song et al., 2018). Most anthropogenic activities surveyed in this study increased substantially by the late 1990s, when HAB frequency started to increase exponentially. It is thus likely anthropogenic activities play crucial roles in the occurrence of HABs in the region. Similar increases in HAB frequencies linked to anthropogenic activities, population, and industrial development were reported as early as 1976–1986 in Tolo Harbor, Hong Kong and 1965–1976 in the Seto Inland Sea of Japan (Lam and Ho, 1989; Okaichi, 1989). We thus speculate that the trend in HAB events in the northern Beibu Gulf is extremely likely to follow HAB patterns in already polluted coastal waters and will therefore further intensify if HAB mitigation and management strategies are not carried out in the near future.

Among the locations, Weizhou Island was the algal blooms hot spot prior to 2010, but blooms occurrences near the island subsequently declined while blooms increased in nearshore waters along the entire Beibu Gulf coast. The volcanic Weizhou Island is a natural source of trace metals such as iron and manganese, and nutrients N and P (Li and Lai, 2007), and the southwest monsoon in the Indian Ocean, which brings high temperatures and strong rainfall to the region, washes terrestrial nutrients into the nearshore waters, which fuels algal blooms. Nutrient-rich seawater passes through the Qiongzhou Strait in the northern Beibu Gulf all year round and elicited a stronger effect on HABs at Weizhou Island than in the bays of Beihai, Qinzhou, and Fangcheng, because summer seawater upwelling delivers abundant nutrients to the sea surface near the island (Yang et al., 2006; Shi, 2014). Most HABs occur at the southern part of Weizhou Island which is the main location for industrial and mariculture activities, and has a dense population that has polluted the coastline (Qiu et al., 2005; He et al., 2013; Li et al., 2014a; Dou et al., 2015). Finally, the nutrient structure in Weizhou Island has changed from N limitation (N:P = 4.1) in the beginning of the 1990s (Wei et al., 2005) to P limitation (N:P = 38.1) in the late 2000s (Sun et al., 2010; He et al., 2013), which may partly explain the decline in HABs after the 2000s.

There were also changes in the ratios of Si:N:P in the northern Beibu Gulf over the study period, the N:P ratio increased from 2.9 to 6.5 in the 1980s to 24.8 to 202.6 in the 1990s in Qinzhou Bay (Wei et al., 2002a), similar trends have been recorded in Tieshan Bay and Lianzhou Bay (Wei et al., 2002b; Lan et al., 2014; Yang et al., 2015). These shifts from N limitation to N abundance, with relative sufficiency of Si, and P limitation (Wu, 2014; Wang F. et al., 2015; Yang et al., 2015) are likely to have changed algal species composition in marine ecosystems (Glibert, 2017). This may explain the recent dominance of *Phaeocystis* blooms in the region, in accordance with the substantial N requirements of *P. globosa* to support its proliferation and sustained blooms (Xu et al., 2003; Zheng, 2014; Gong et al., 2018).

Temporal shifts in dominant HAB species driven by nutrient conditions have been widely seen in Chinese waters. Changjiang River estuary, the most notable HAB area in China, has experienced an obvious change of dominant blooming microalgae from diatoms to noxious or toxic dinoflagellates in the last 30 years (Yang et al., 2015; Yu et al., 2017). This shift was thought to be largely driven by the increasing ratio of DIN:P and high DIN, which increased 4-fold from 20.5 μM in the 1960s to 80.0 μM in the 2000s (Ye et al., 2004; Wang and Cao, 2012; Yu et al., 2017). During 2009–2016, the proportion of dinoflagellate blooms has also risen to as high as 79% on the south China coast, induced by changes in seawater temperature, DIN, and P concentration (Yi et al., 2018). Changes in phytoplankton communities may also relate to climate change (Hallegraeff, 2010; Wells and Karlson, 2018), which needs to be investigated in the future for the northern Beibu Gulf.

The haptophyte *Phaeocystis* forms massive HABs in many parts of the world, from tropical and subtropical to Arctic and Antarctic oceans (Long et al., 2007; Smith et al., 2017). This bloom is recognized both as a nuisance and as an ecologically important phytoplankton, with production of haemolytic substances in relation to the risk of marine life and human health, as well as dimethylsulfide (DMS) involved in the biological regulation of the climate (Medlin and Zingone, 2007; Wang S. et al., 2015). So far, six *Phaeocystis* species have been clearly defined, and only three of them, *P. globosa, P. pouchetii*, and *P. antarctica*, were described to date as bloom forming species (Schoemann et al., 2005; Medlin and Zingone, 2007). However, in Chinese seawaters *P. globosa* is the only bloom causing organism within this genus to date, with its blooms first documented from Oct 1997-Feb 1998 in southeast China, covering thousands of km^2^ in the coastal waters of Fujian and Guangdong (Chen et al., 1999; Qi et al., 2002). It then frequently appeared in southern coastal areas (Hainan, Hong Kong, and Guangdong), and Bohai and Yellow River estuaries from 2000 to 2009 (Qi et al., 2002; Li et al., 2012). Blooms of *P. globosa* started to occur from 2011 in the northern Beibu Gulf and have continued breaking out in recent years (Table 1). *Phaeocystis* has a complex polymorphic life cycle alternating between flagellated cells and colonies enclosed in an exopolysaccharide matrix (Rousseau et al., 2013), protecting it from predators, enhanced UVB, and viral and bacterial infections (Brussaard et al., 2005; Kennedy et al., 2012). Interestingly, *Phaeocystis* develops blooms in the colonial form, particularly in waters with abundant nitrates, phosphate, and urea (Schoemann et al., 2005; Wang et al., 2007; Liang et al., 2018). In addition to the advantages of colony morphology, another competitive advantage of *P. globosa* in the northern Beibu Gulf may be its environmental adaptations, such as high tolerance of varied environmental light, preference for high temperatures and flexible nutrient uptake strategy (Schoemann et al., 2005; Xu et al., 2017).

Both abiotic and biotic factors affect the formation and duration of HAB events (Smith et al., 1999; Lewitus et al., 2012; Smetacek, 2012; Smida et al., 2012). In this study, about 65.7% of HAB variation was explained by human activity factors, environmental factors, and industrial development, indicating that these parameters are dominant determinants of HAB formation. Our findings support the previous viewpoint that abiotic factors are the main drivers affecting algal bloom formation and community structure (Worden et al., 2015). On the other hand, we note that 34.3% of HAB variation was not explained by the data measured, which indicates that other abiotic (water chemistry and hydrodynamics) and/or biotic (algal species interactions, viral lysis, reactive oxygen species abundance, and fungal and zooplankton predation) factors that were not included in this analysis may be important drivers of HAB events (Sun et al., 2017; Karasiewicz et al., 2018; Zhang et al., 2018). Further investigation of the interactions among algae, bacteria, viruses, and ciliates, particularly their network relationship over longer time scales, is needed to better characterize the underlying mechanisms driving the increases in HAB events and phytoplankton community variation in the northern Beibu Gulf.

## Conclusion

Algal blooms in the northern Beibu Gulf have intensified over the past three decades, likely as a result of anthropogenic influences. Continuing economic and industrial development, as well as a rising human population in the region, will lead to further increases in HAB events in the future. Thus, HAB mitigation strategies should include: (1) an increase in the number of buoys and automatic monitoring stations along the northern Beibu Gulf coast; (2) the development of satellite remote sensing, *in situ* cell observations, and computer modeling to predict bloom events; (3) reductions in nutrient discharge and/or improved management for N/P removal; and, (4) the creation of a science-based ecological management plan to perform the ecophysiological and toxicological characterization of regional algal bloom species. There should be government-level efforts to enrich database records of HAB events and analyze the key environmental drivers of HABs to improve the sustainable development of this region.

## Author Contributions

YX and TZ collect the data and designed the experiments. YX and JZ contributed to the analyses work. The manuscript was first written by YX and TZ, and then improved by YX and JZ.

### Conflict of Interest Statement

The authors declare that the research was conducted in the absence of any commercial or financial relationships that could be construed as a potential conflict of interest.
