# Supplementary material for: Historical Occurrence of Algal Blooms in the Northern Beibu Gulf of China and Implications for Future Trends
Source: Front Microbiol. 2019 Mar 13;10:451. doi: 10.3389/fmicb.2019.00451 (PMC6424905; doi:10.3389/fmicb.2019.00451)
Supplement: Supplementary file 6 [file Data_Sheet_1.PDF]

Supplement 1. Population (10^4 persons) for Nanning, Beihai, Qinzhou and Fangchenggang from 1988-2015. Data originated from Guangxi Statistical Yearbook.

Year	Nanning	Beihai	Qinzhou	Fangchenggang	References
1988	103.14	19.01	99.86	1.35	1989 Guangxi Statistical Yearbook, pp.450
1989	105.00	19.47	101.77	1.48	1989 Guangxi Statistical Yearbook, pp.419
1990	107.00	20.02	103.53	1.58	1991 Guangxi Statistical Yearbook, pp.428
1991	108.58	20.56	105.12	1.79	1992 Guangxi Statistical Yearbook, pp.469
1992	111.57	21.91	106.44	2.76	1993 Guangxi Statistical Yearbook, pp.301
1993	264.61	130.33	107.50	71.80	1994 Guangxi Statistical Yearbook, pp.365
1994	268.66	132.00	297.56	72.62	1995 Guangxi Statistical Yearbook, pp.369
1995	273.19	134.09	300.97	73.75	1996 Guangxi Statistical Yearbook, pp.439
1996	277.92	135.42	304.63	74.78	1997 Guangxi Statistical Yearbook, pp.399
1997	281.20	136.49	308.37	75.54	1998 Guangxi Statistical Yearbook, pp.379
1998	284.63	137.79	311.43	76.08	1999 Guangxi Statistical Yearbook, pp.375
1999	285.87	139.52	314.82	77.09	2000 Guangxi Statistical Yearbook, pp.357
2000	293.34	141.71	326.86	77.84	2001 Guangxi Statistical Yearbook, pp.333
2001	294.40	144.70	329.90	77.70	2002 Guangxi Statistical Yearbook, pp.385
2002	297.71	145.49	333.44	78.31	2003 Guangxi Statistical Yearbook, pp.395
2003	641.67	146.77	336.74	78.85	2004 Guangxi Statistical Yearbook, pp.441
2004	648.85	147.87	344.01	79.84	2005 Guangxi Statistical Yearbook, pp.437
2005	659.54	149.24	341.10	79.82	2006 Guangxi Statistical Yearbook, pp.475
2006	671.89	152.06	348.56	82.21	2007 Guangxi Statistical Yearbook, pp.463
2007	683.51	156.32	356.00	83.32	2008 Guangxi Statistical Yearbook, pp.467
2008	691.69	157.72	364.51	84.76	2009 Guangxi Statistical Yearbook, pp.431
2009	701.30	158.97	326.18	85.28	2010 Guangxi Statistical Yearbook, pp.457
2010	666.16	153.93	307.97	86.69	2011 Guangxi Statistical Yearbook, pp.485

2011	673.40	155.44	310.96	87.84	2012 Guangxi Statistical Yearbook, pp.545
2012	679.08	157.20	313.33	88.69	2013 Guangxi Statistical Yearbook, pp.537
2013	685.37	159.02	315.92	89.90	2014 Guangxi Statistical Yearbook, pp.509
2014	691.38	160.37	318.06	90.80	2015 Guangxi Statistical Yearbook, pp.504
2015	698.61	162.57	320.93	91.84	2016 Guangxi Statistical Yearbook, pp.51
