# Supplementary material for: Historical Occurrence of Algal Blooms in the Northern Beibu Gulf of China and Implications for Future Trends
Source: Front Microbiol. 2019 Mar 13;10:451. doi: 10.3389/fmicb.2019.00451 (PMC6424905; doi:10.3389/fmicb.2019.00451)
Supplement: Supplementary file 7 [file Data_Sheet_2.PDF]

Supplement 2. Gross industrial output value (Above designated size, at current prices, 10^8 Yuan) for Nanning, Beihai, Qinzhou and Fangchenggang from 1988-2015. Data originated from Guangxi Statistical Yearbook.

Year	Nanning	Beihai	Qinzhou	Fangchenggang	References
1988	33.0029	4.9269	4.0242	0.0573	1989 Guangxi Statistical Yearbook, pp.394
1989	41.4888	5.9289	4.6714	0.2207	1990 Guangxi Statistical Yearbook, pp.420
1990	44.7989	7.0703	5.2387	0.0742	1991 Guangxi Statistical Yearbook, pp.456
1991	54.2461	8.9019	6.3705	0.1723	1992 Guangxi Statistical Yearbook, pp.452
1992	59.5117	11.9501	9.7909	0.3328	1993 Guangxi Statistical Yearbook, pp.301
1993	100.5613	49.4770	17.4487	8.8773	1994 Guangxi Statistical Yearbook, pp.366
1994	134.1785	70.6105	54.7555	17.2770	1995 Guangxi Statistical Yearbook, pp.370
1995	175.9443	79.1767	56.1677	26.4884	1996 Guangxi Statistical Yearbook, pp.431
1996	158.1367	89.6926	73.5351	29.7162	1997 Guangxi Statistical Yearbook, pp.391
1997	169.6077	105.2775	85.7009	38.2420	1998 Guangxi Statistical Yearbook, pp.374
1998	182.4639	86.9333	63.1300	37.0826	1999 Guangxi Statistical Yearbook, pp.369
1999	187.0681	88.0241	62.0321	38.5070	2000 Guangxi Statistical Yearbook, pp.353
2000	126.8047	43.8750	18.8478	14.6349	2001 Guangxi Statistical Yearbook, pp.361
2001	133.7751	47.6529	21.2841	16.8882	2002 Guangxi Statistical Yearbook, pp.413
2002	149.1185	48.0888	25.4003	32.7629	2003 Guangxi Statistical Yearbook, pp.422
2003	208.3800	55.0800	31.1000	51.0900	2004 Guangxi Statistical Yearbook, pp.475
2004	263.6500	74.7300	38.6600	66.3700	2005 Guangxi Statistical Yearbook, pp.473
2005	370.1800	102.7900	74.9100	86.2800	2006 Guangxi Statistical Yearbook, pp.490
2006	495.4800	132.5000	120.7600	114.6500	2007 Guangxi Statistical Yearbook, pp.480
2008	864.9600	253.0500	256.1500	278.6600	2009 Guangxi Statistical Yearbook, pp.448
2009	981.6500	230.8800	271.4500	342.6500	2010 Guangxi Statistical Yearbook, pp.474
2010	1285.4000	103.0000	481.7400	453.9300	2011 Guangxi Statistical Yearbook, pp.500
2011	1725.2900	535.2700	940.5600	635.5600	2012 Guangxi Statistical Yearbook, pp.560

2012	2109.3300	1026.2000	1093.3600	767.2300	2013 Guangxi Statistical Yearbook, pp.554
2013	2557.1300	1284.9100	1130.5800	964.4100	2014 Guangxi Statistical Yearbook, pp.527
2014	2856.6300	1595.5000	1291.4400	1141.0800	2015 Guangxi Statistical Yearbook, pp.522
2015	3237.0606	1844.8693	1358.4674	1300.8998	2016 Guangxi Statistical Yearbook, pp.528
