# Supplementary material for: Historical Occurrence of Algal Blooms in the Northern Beibu Gulf of China and Implications for Future Trends
Source: Front Microbiol. 2019 Mar 13;10:451. doi: 10.3389/fmicb.2019.00451 (PMC6424905; doi:10.3389/fmicb.2019.00451)
Supplement: Supplementary file 8 [file Data_Sheet_3.PDF]

Supplement 3. Gross domestic product (10^8 Yuan) for Nanning, Beihai, Qinzhou and Fangchenggang from 1988-2015. Data originated from Guangxi Statistical Yearbook.

Year	Nanning	Beihai	Qinzhou	Fangchenggang	References
1988	24.3725	5.0030	6.6538	-	1989 Guangxi Statistical Yearbook, pp.383
1989	30.0593	5.0054	7.3914	0.7151	1990 Guangxi Statistical Yearbook, pp.407
1990	34.7625	6.7065	9.1073	0.8147	1991 Guangxi Statistical Yearbook, pp.455
1991	38.1114	7.4980	11.3097	1.0492	1992 Guangxi Statistical Yearbook, pp.451
1992	62.4798	12.5364	16.4095	2.2328	1993 Guangxi Statistical Yearbook, pp.301
1993	94.8987	55.1627	22.3069	17.3471	1994 Guangxi Statistical Yearbook, pp.365
1994	136.1000	76.8600	64.4500	26.0500	1995 Guangxi Statistical Yearbook, pp.369
1995	174.8934	89.9274	92.4853	32.0649	1996 Guangxi Statistical Yearbook, pp.429
1996	202.1974	97.0487	112.3179	39.6995	1997 Guangxi Statistical Yearbook, pp.389
1997	231.5969	109.4059	127.3312	48.2275	1998 Guangxi Statistical Yearbook, pp.373
1998	257.5733	102.6208	119.1104	51.9722	1999 Guangxi Statistical Yearbook, pp.367
1999	274.5506	107.6779	123.8784	54.6187	2000 Guangxi Statistical Yearbook, pp.351
2000	294.3000	113.6800	132.3600	57.5100	2001 Guangxi Statistical Yearbook, pp.333
2001	324.7900	123.4400	144.0900	62.6500	2002 Guangxi Statistical Yearbook, pp.385
2002	356.0700	134.3900	154.6600	69.2800	2003 Guangxi Statistical Yearbook, pp.395
2003	502.5300	140.1400	155.3300	71.9800	2004 Guangxi Statistical Yearbook, pp.441
2004	588.8600	161.8900	174.6500	84.6000	2005 Guangxi Statistical Yearbook, pp.437
2005	723.3600	181.6200	205.5200	94.7700	2006 Guangxi Statistical Yearbook, pp.475
2006	870.1500	199.6400	245.0700	119.6100	2007 Guangxi Statistical Yearbook, pp.463
2007	1069.0100	246.5800	303.9200	159.2800	2008 Guangxi Statistical Yearbook, pp.467
2008	1316.2100	313.8800	377.4200	212.1800	2009 Guangxi Statistical Yearbook, pp.431
2009	1524.7100	321.0600	396.1800	251.0400	2010 Guangxi Statistical Yearbook, pp.457
2010	1800.2600	404.4100	520.6700	320.4200	2011 Guangxi Statistical Yearbook, pp.487

2011	2211.4400	498.3100	646.6500	413.7700	2012 Guangxi Statistical Yearbook, pp.547
2012	2503.1800	630.0900	691.3200	443.9900	2013 Guangxi Statistical Yearbook, pp.537
2013	2803.5400	735.0000	753.7400	525.1500	2014 Guangxi Statistical Yearbook, pp.509
2014	3148.3200	856.5400	854.9600	588.8900	2015 Guangxi Statistical Yearbook, pp.504
2015	3410.0800	891.9400	944.4200	620.7100	2016 Guangxi Statistical Yearbook, pp.524
