# Supplementary material for: Historical Occurrence of Algal Blooms in the Northern Beibu Gulf of China and Implications for Future Trends
Source: Front Microbiol. 2019 Mar 13;10:451. doi: 10.3389/fmicb.2019.00451 (PMC6424905; doi:10.3389/fmicb.2019.00451)
Supplement: Supplementary file 9 [file Data_Sheet_4.PDF]

Supplement 4. Consumption of chemical fertilizers (Pure quantity, 10^4 tons) for Nanning, Beihai, Qinzhou and Fangchenggang from 1988-2015. Data originated from Guangxi Statistical Yearbook.

Year	Nanning	Beihai	Qinzhou	Fangchenggang	References
1988	0.795	0.1012	1.6661	0.0003	1989 Guangxi Statistical Yearbook, pp.385
1989	0.8322	0.1918	1.6047	0.0004	1990 Guangxi Statistical Yearbook, pp.420
1990	1.0496	0.3971	2.1196	0.0008	1991 Guangxi Statistical Yearbook, pp.456
1991	1.3300	0.4328	2.6099	0.0012	1992 Guangxi Statistical Yearbook, pp.452
1993	9.3934	4.6270	3.8544	2.2613	1994 Guangxi Statistical Yearbook, pp.365
1994	10.9628	4.5837	9.5241	2.3941	1995 Guangxi Statistical Yearbook, pp.369
1995	13.1458	5.3816	12.5721	2.3189	1996 Guangxi Statistical Yearbook, pp.430
1996	15.0788	5.4962	13.1722	2.3407	1997 Guangxi Statistical Yearbook, pp.390
1997	16.0461	5.7715	14.2988	2.6324	1998 Guangxi Statistical Yearbook, pp.374
1998	15.9756	5.9047	16.1685	2.7206	1999 Guangxi Statistical Yearbook, pp.368
1999	15.0159	6.0697	17.0122	2.8289	2000 Guangxi Statistical Yearbook, pp.352
2000	15.2000	5.3021	17.0270	2.8591	2001 Guangxi Statistical Yearbook, pp.361
2001	16.8093	5.4603	18.5881	3.0388	2002 Guangxi Statistical Yearbook, pp.413
2002	16.6703	5.6713	19.0980	3.1728	2003 Guangxi Statistical Yearbook, pp.422
2003	31.3800	5.7000	19.3900	3.2400	2004 Guangxi Statistical Yearbook, pp.475
2004	34.4200	5.7200	20.2000	3.2600	2005 Guangxi Statistical Yearbook, pp.453
2005	34.8700	5.8600	21.2600	3.6400	2006 Guangxi Statistical Yearbook, pp.501
2006	36.6600	6.0400	22.8700	3.8200	2007 Guangxi Statistical Yearbook, pp.480
2008	40.5400	6.2900	24.0300	4.3100	2009 Guangxi Statistical Yearbook, pp.448
2010	43.0000	6.2000	24.9000	5.0000	2011 Guangxi Statistical Yearbook, pp.500
2011	44.0000	6.2900	25.5000	4.9000	2012 Guangxi Statistical Yearbook, pp.560
2012	44.3400	6.3300	25.7600	5.2600	2013 Guangxi Statistical Yearbook, pp.554
2013	45.5000	6.5100	26.3200	6.2700	2014 Guangxi Statistical Yearbook, pp.527

2014	46.8200	6.5000	26.7400	6.4800	2015 Guangxi Statistical Yearbook, pp.523
2015	48.5100	6.6000	25.3000	6.2200	2016 Guangxi Statistical Yearbook, pp.528
