# Supplementary material for: Historical Occurrence of Algal Blooms in the Northern Beibu Gulf of China and Implications for Future Trends
Source: Front Microbiol. 2019 Mar 13;10:451. doi: 10.3389/fmicb.2019.00451 (PMC6424905; doi:10.3389/fmicb.2019.00451)
Supplement: Supplementary file 10 [file Data_Sheet_5.PDF]

Supplement 5. Guangxi seawater cultured areas (10^3 hectares) from 1978-2006. Data originated from Guangxi Statistical Yearbook.

Year	Seawater cultured areas	References
1978	1.9	1994 Guangxi Statistical Yearbook, pp.220
1980	1.6	1994 Guangxi Statistical Yearbook, pp.220
1985	1.9	1994 Guangxi Statistical Yearbook, pp.220
1990	5.5	1994 Guangxi Statistical Yearbook, pp.220
1991	8.7	1994 Guangxi Statistical Yearbook, pp.220
1992	14.6	1994 Guangxi Statistical Yearbook, pp.220
1993	17.2	1994 Guangxi Statistical Yearbook, pp.220
1994	29.9	1995 Guangxi Statistical Yearbook, pp.206
1995	41.0	1996 Guangxi Statistical Yearbook, pp.239
1996	48.4	1997 Guangxi Statistical Yearbook, pp.224
1997	52.2	1998 Guangxi Statistical Yearbook, pp.220
1998	55.6	1999 Guangxi Statistical Yearbook, pp.195
1999	58.6	2000 Guangxi Statistical Yearbook, pp.199
2000	61.4	2001 Guangxi Statistical Yearbook, pp.201
2001	61.3	2002 Guangxi Statistical Yearbook, pp.236
2002	61.7	2003 Guangxi Statistical Yearbook, pp.255
2003	62.2	2004 Guangxi Statistical Yearbook, pp.281
2004	61.9	2005 Guangxi Statistical Yearbook, pp.283
2005	62.0	2006 Guangxi Statistical Yearbook, pp.311
2006	63.8	2007 Guangxi Statistical Yearbook, pp.295
