# Supplementary material for: Historical Occurrence of Algal Blooms in the Northern Beibu Gulf of China and Implications for Future Trends
Source: Front Microbiol. 2019 Mar 13;10:451. doi: 10.3389/fmicb.2019.00451 (PMC6424905; doi:10.3389/fmicb.2019.00451)
Supplement: Supplementary file 11 [file Data_Sheet_6.PDF]

Supplement 6. Guangxi artificially cultured products (10^4 tons) from 1978-2015. Data originated from Guangxi Statistical Yearbook.

Year	Artificially cultured products	References
1978	0.0438	1994 Guangxi Statistical Yearbook, pp.220
1980	0.0633	1994 Guangxi Statistical Yearbook, pp.220
1985	0.0698	1994 Guangxi Statistical Yearbook, pp.220
1990	0.3348	1994 Guangxi Statistical Yearbook, pp.220
1991	1.1919	1994 Guangxi Statistical Yearbook, pp.220
1992	2.3725	1994 Guangxi Statistical Yearbook, pp.220
1993	4.5358	1994 Guangxi Statistical Yearbook, pp.220
1994	7.5964	1995 Guangxi Statistical Yearbook, pp.206
1995	14.7514	1996 Guangxi Statistical Yearbook, pp.239
1996	12.1823	1997 Guangxi Statistical Yearbook, pp.224
1997	49.8209	1998 Guangxi Statistical Yearbook, pp.220
1998	57.3641	1999 Guangxi Statistical Yearbook, pp.195
1999	66.5703	2000 Guangxi Statistical Yearbook, pp.199
2000	70.6088	2001 Guangxi Statistical Yearbook, pp.201
2001	74.6738	2002 Guangxi Statistical Yearbook, pp.236
2002	80.1200	2003 Guangxi Statistical Yearbook, pp.255
2003	83.6326	2004 Guangxi Statistical Yearbook, pp.281
2004	85.8562	2005 Guangxi Statistical Yearbook, pp.283
2005	89.3795	2006 Guangxi Statistical Yearbook, pp.311
2006	92.9735	2007 Guangxi Statistical Yearbook, pp.295
2007	76.3645	2008 Guangxi Statistical Yearbook, pp.300
2008	77.5866	2009 Guangxi Statistical Yearbook, pp.290
2009	82.2505	2010 Guangxi Statistical Yearbook, pp.346
2010	87.7408	2013 Guangxi Statistical Yearbook, pp.362
2011	92.3804	2013 Guangxi Statistical Yearbook, pp.362
2012	97.5577	2013 Guangxi Statistical Yearbook, pp.362
2013	105.5626	2014 Guangxi Statistical Yearbook, pp.332
2014	109.0975	2015 Guangxi Statistical Yearbook, pp.316
2015	114.2166	2016 Guangxi Statistical Yearbook, pp.321
