# Supplementary material for: Historical Occurrence of Algal Blooms in the Northern Beibu Gulf of China and Implications for Future Trends
Source: Front Microbiol. 2019 Mar 13;10:451. doi: 10.3389/fmicb.2019.00451 (PMC6424905; doi:10.3389/fmicb.2019.00451)
Supplement: Supplementary file 12 [file Data_Sheet_7.PDF]

Supplement 7. Guangxi seawater aquatic products (10^4 tons) from 1978-2015. Data originated from Guangxi Statistical Yearbook.

Year	Seawater aquatic products	References
1978	8.5900	1992 Guangxi Statistical Yearbook, pp.242
1979	6.3400	1992 Guangxi Statistical Yearbook, pp.242
1980	7.9200	1992 Guangxi Statistical Yearbook, pp.242
1981	8.2100	1992 Guangxi Statistical Yearbook, pp.242
1982	10.3800	1992 Guangxi Statistical Yearbook, pp.242
1983	11.6400	1992 Guangxi Statistical Yearbook, pp.242
1984	11.5000	1992 Guangxi Statistical Yearbook, pp.242
1985	12.1900	1992 Guangxi Statistical Yearbook, pp.242
1986	13.1400	1992 Guangxi Statistical Yearbook, pp.242
1987	15.1400	1992 Guangxi Statistical Yearbook, pp.242
1988	16.4300	1992 Guangxi Statistical Yearbook, pp.242
1989	17.7300	1992 Guangxi Statistical Yearbook, pp.242
1990	20.2700	1992 Guangxi Statistical Yearbook, pp.242
1991	23.4200	1992 Guangxi Statistical Yearbook, pp.242
1992	28.4350	1993 Guangxi Statistical Yearbook, pp.158
1993	34.8364	1994 Guangxi Statistical Yearbook, pp.220
1994	47.1877	1995 Guangxi Statistical Yearbook, pp.206
1995	64.5706	2007 Guangxi Statistical Yearbook, pp.295
1996	72.0932	1998 Guangxi Statistical Yearbook, pp.220
1997	128.0870	1998 Guangxi Statistical Yearbook, pp.220
1998	146.1470	2000 Guangxi Statistical Yearbook, pp.199
1999	155.4260	2000 Guangxi Statistical Yearbook, pp.199
2000	159.4505	2007 Guangxi Statistical Yearbook, pp.295
2001	163.9807	2002 Guangxi Statistical Yearbook, pp.236
2002	166.4169	2004 Guangxi Statistical Yearbook, pp.281
2003	168.7368	2004 Guangxi Statistical Yearbook, pp.281
2004	171.2294	2007 Guangxi Statistical Yearbook, pp.295
2005	173.9581	2007 Guangxi Statistical Yearbook, pp.295
2006	176.9508	2007 Guangxi Statistical Yearbook, pp.295
2007	143.3236	2008 Guangxi Statistical Yearbook, pp.300
2008	144.0596	2010 Guangxi Statistical Yearbook, pp.346
2009	148.5484	2010 Guangxi Statistical Yearbook, pp.346
2010	154.0362	2017 Guangxi Statistical Yearbook, pp.333
2011	158.9085	2017 Guangxi Statistical Yearbook, pp.333
2012	164.3851	2017 Guangxi Statistical Yearbook, pp.333
2013	170.7060	2017 Guangxi Statistical Yearbook, pp.333
2014	174.1574	2017 Guangxi Statistical Yearbook, pp.333
2015	179.4194	2017 Guangxi Statistical Yearbook, pp.333
