# Supplementary material for: Historical Occurrence of Algal Blooms in the Northern Beibu Gulf of China and Implications for Future Trends
Source: Front Microbiol. 2019 Mar 13;10:451. doi: 10.3389/fmicb.2019.00451 (PMC6424905; doi:10.3389/fmicb.2019.00451)
Supplement: Supplementary file 13 [file Data_Sheet_8.PDF]

Supplement 8. Guangxi marine related GDP (10^9 Yuan) from 2007-2016. Data originated from Guangxi Marine Economic Statistics Bulletin, http://www.gxoa.gov.cn/zwgk_list?id=15. Accessed Dec 21, 2018 (in Chinese).

Year	2007	2008	2009	2010	2011	2012	2013	2014	2015	2016
Marine related GDP	320	442	443	549	654	693	899	926	1098	1233
