# Supplementary material for: Historical Occurrence of Algal Blooms in the Northern Beibu Gulf of China and Implications for Future Trends
Source: Front Microbiol. 2019 Mar 13;10:451. doi: 10.3389/fmicb.2019.00451 (PMC6424905; doi:10.3389/fmicb.2019.00451)
Supplement: Supplementary file 14 [file Data_Sheet_9.PDF]

Supplement 9. Fishing boats in the northern Beibu Gulf of China from 2001-2010. Data originated from Lan and Li (2013).

Year	Number of fishing boat	Number of fishing boat through harbor	Effluent by boat
2001	12131	5308	183.65
2002	13034	6202	204.99
2003	13853	16418	369.20
2004	12870	9165	257.51
2005	12330	9692	261.93
2006	15214	10029	282.24
2007	15022	22233	468.57
2008	14523	26640	532.25
2009	14114	28152	552.10
2010	12506	37665	685.09

Lan, W. L., and Li, T. S. (2013). Problems and countermeasures of marine ecosystem Qinzhou Bay. *Environ. Sci. Manag.* 38(1), 118-146 (in Chinese).
