# Supplementary material for: Historical Occurrence of Algal Blooms in the Northern Beibu Gulf of China and Implications for Future Trends
Source: Front Microbiol. 2019 Mar 13;10:451. doi: 10.3389/fmicb.2019.00451 (PMC6424905; doi:10.3389/fmicb.2019.00451)
Supplement: Supplementary file 15 [file Data_Sheet_10.pdf]

Supplement 10. Areas of five classification of summer seawater quality between 2010-2016 in the northern Beibu Gulf (km^2). Seawater quality is categorized from I–IV, where Grade Ⅰ is defined as high quality seawater for ocean fishing, marine nature reserves that contains rare and endangered marine organism, while Grade Ⅳ represents the lowest quality seawater used for harbor and ocean engineering operations. <Grade Ⅰ = Grade Ⅱ + Grade Ⅲ+ Grade Ⅳ+ < Grade Ⅳ.

Year	Grade Ⅱ	Grade Ⅲ	Grade Ⅳ	< Grade Ⅳ	< Grade Ⅰ	References
2010	133	2601	81	320	3135	[1]
2011	345	581	39	167	1132	[1]
2012	470	1530	320	300	2620	[1]
2013	1209	526	108	838	2681	[1]
2014	2650	306	174	466	3596	[1]
2015	787	2787	261	562	4397	[2]
2016	2207	687	282	10	3186	[3]

[1] Department of Ocean and Fisheries of Guangxi Zhuang Autonomous Region (2015). 2014 marine environmental quality bulletin of Guangxi. Available at: http://www.gxoa.gov.cn/gxhyj_haiyanggongbao/2015/06/16/74446b624f1b4746b3f14e94910a3846.html. Accessed Dec 21, 2018 (in Chinese).

[2] Department of Ocean and Fisheries of Guangxi Zhuang Autonomous Region (2016). 2015 marine environmental quality bulletin of Guangxi. Available at: http://www.gxoa.gov.cn/gxhyj_haiyanggongbao/2016/05/31/19c0a564a83146fd8cb99b8c8ebc5a88.html. Accessed Dec 21, 2018 (in Chinese).

[3] Department of Ocean and Fisheries of Guangxi Zhuang Autonomous Region (2017). 2016 marine environmental quality bulletin of Guangxi. Available at: http://www.gxoa.gov.cn/gxhyj_haiyanggongbao/2017/05/24/49637df706fc42e1be24a01ebe7a97ce.html. Accessed Dec 21, 2018 (in Chinese).
